# Contra Consilium Medicinae: A Strange Malady Affecting Indian Healthcare!!

**DOI:** 10.5005/jp-journals-10071-23131

**Published:** 2019-03

**Authors:** Rahul A Pandit, Atul P Kulkarni

**Affiliations:** 1 Department of Intensive Care, Fortis Hospital, Mumbai, India; 2 Department of Anaesthesiology, Critical Care and Pain, Tata Memorial Hospital, Homi Bhabha National Institute, Mumbai, India

## Abstract

**How to cite this article:** Pandit RA, Kulkarni AP. Contra Consilium Medicinae: A Strange Malady Affecting Indian Healthcare!!. Indian J Crit Care Med 2019;23(3):113-114.

Contra cosilium medicinae, i.e. the patient leaving the hospital before the treating doctor recommends discharge, is a phenomena seen worldwide^1^. It is known by various synonyms: DAMA-discharge against medical advice, LAMA-leave against medical advice, signing out by selfs etc. However, multidisciplinary, prospective studies have been very few in this group of patients. It is also important to understand that the outcome of these patients remains unknown more often than not, as they are lost to follow-up. In group of patients who underwent a percutaneous coronary intervention and were discharged against medical advice, they were twice as likely to be readmitted and possibly have a poor outcome^2^. There is a significant ethical, legal and clinical challenge in managing these patients. While it may seem that the responsibility has been shifted on to the patients and their family's shoulders, it is often legally nebulous with uncertain consequences. The phenomena seems to be more prevalent in countries where healthcare delivery is not paid for by state. In countries where the state pays for healthcare of its citizens, there seems to be a lower incidence of DAMA^3^.

In this issue, Mahajan, and Paul et al.^4,5^ report findings of their studies about this important issue. The first study is a retrospective analysis of patients Leaving against medical advice in a tertiary care teaching hospital. The study highlights that over a period of one year 3227 (4.95%) patients took LAMA from the hospital. The mean age was 47 years, but patients ages ranged from newborn to 103 years of age. Surprisingly there was a male predominance (2:1 ratio of male: female in patients taking DAMA).

The results also sound a cautionary note that more than 58% of patients or their families decided for DAMA when the patient was on mechanical ventilation. Most common diagnosis was trauma and one fifth patients had a poor or guarded prognosis explained. The authors have further looked into the socio-economic background of these people. With a well-established Kuppuswamy's scale, the authors bring forward the finding that almost 38% of patients were unemployed and 23% were people of simple means who probably represented the weaker socio-economic strata of population. The authors need to be commended for this important retrospective analysis, which brings forward a very important aspect of our healthcare delivery which is ‘cost’. This being a single center study, it may represent a local problem.

Paul and Gautam et al.^5^ conducted a National Survey of Doctors, mainly anesthesiologist and intensivist, to get a better perception of geographical variability, burden of this strange malady across the nation and importantly the doctors perception and understanding of reasons for DAMA. The self-developed 22 items and four-section questionnaire covered patients' demographics, types of hospital, doctor's perception about factors related to DAMA and doctor's perception about the reasons for DAMA.

The authors had a mixed response to the online survey. Around 50% of doctors did not answer all the questions. Doctors from some states like Maharashtra and Tamil Nadu were more proactive in answering the survey. Similarly, private and corporate hospital doctors with more than 100 beds hospital were the major responders.

The point of concern in this survey is the fact that majority of patients (94%) went DAMA from acute care areas (62% from ICU, 32% from Emergency department) alluding to the fact that patients and families requesting DAMA were doing so more from acute care areas^5^.

Authors have done well to bring forward the real problems faced by Indian healthcare scenario, where cost plays a major role in delivery of healthcare. Lack of health Insurance was cited as the most common perception (90%) by most responders. This issue has been exensively addressed by Naderi and Acerra et al.^6^ previsouly as well.

The two studies bring out some very important apects in effective delivery of healthcare in India.^4,5^ The first study despite its single center can be extrapolated to most of the tertiary care institutes across the country.^4^ The clinical dilemma faced by doctors, when their patients seek DAMA, is not because of ineffective care, but more due to cost constrains. Also the high risk of transferring the critical patients poses a great challenge, unfortunately the study was not designed to look in to this critical aspect. The lack of standardized ambulance services across the country compounds the problem by several fold. Also it is clearly established that patients who seek LAMA/DAMA tend to have poor outcomes, quite often they seek readmission to similar or public run hospitals^6,7^, but precious time is lost which could have been utilized in providing urgent medical care.

The second study brings forward the perceptions of doctors about DAMA/LAMA^5^. It clearly highlights the cost as a major issue. The lack of clarity in cases of medical futility and options (in the legal sense) and grey areas in implementing end of life care also come forth strongly in this survery. It seems obvious that doctors from developing countries may have innovated DAMA/LAMA as a practice. However, bringing forward a uniform protocol for end of life care will make dying process full of dignity and without distress.

The key difference seen in both the studies in India and developed countries is the fact that in developing countries cost seems to be the major factor for DAMA and in developed world alcohol and substance addiction seem to the common reason for DAMA. This contrasting difference is only brought forward because of these two studies, as most of the evidence surrounding DAMA/LAMA comes from developed world^2,7^.

More prospective multicenter studies are required evaluating this unique problem facing acute care practitioners in our country. Acute care costs is an unsolvable problem, till we get a state-paid healthcare system, which is not pragmatic at this juncture. We are also a long way away from universal insurance cover. This leaves us with very few options, most of them unsurmountable. Early identification of the patients who will later request DAMA/LAMA and early and safe transfer to hospitals which will provide cheaper healthcare seems like a pipe dream. Transitioning care safely may be done, but costs preclude this option. For patients to whom medical care looks futile, offering counseling early, facilitating terminal home care may be possible. It is time for all stakeholders to think about this strange problem that affects a large proportion of our acute care patients and search for a solution^8–10^.

## References

[1] Alfandre DJ, (2009;). “I'm going home” discharge against medical advice.. Mayo Clin Proc..

[2] Kwok CS,, Bell M (2018). Discharge against medical advice after percutaneous coronary intervention in the United States.. JACC Cardiovasc Interv..

[3] Jeong J,, Song KJ (2016). The association between acute alcohol consumption and discharge against medical advice of injured patients in the ED.. Am J Emerg Med..

[4] Mahajan RK,, Gautam PL (2019,). Retrospective evaluation of patients leaving against medical advice in a tertiary care teaching hospital.. IJCCM.

[5] Paul G,, Gautam PL (2019;). Patients leaving against medical advice-A national survey.. Indian J of Crit Care Med.

[6] Naderi S,, Acerra J R (2014;). Patients in a private hospital in India leave the emergency department against medical advice for financial reasons.. International Journal of Emergency Medicine..

[7] Nasir AA,, Babalola OM. (2008;). Clinical spectrum of discharges against medical advice in a developing country. Indian J.. Surg.

[8] Hwang S W,, Li J (2003;). “What happens to patients who leave hospital against medical advice?”. Canadian Medical Association Journal..

[9] Southern WN,, Nahvi S (2012;). Increased risk of mortality and readmission among patients discharged against medical advice.. Am J Med..

[10] Ti L,, Milloy M-J Factors associated with leaving hospital against medical advice among people who use illicit drugs in vancouver, Canada.. PLoS ONE.

